# Gender Comparisons of Physical Fitness Indexes in Inner Mongolia Medical Students in China

**DOI:** 10.5539/gjhs.v7n1p220

**Published:** 2014-08-31

**Authors:** Wenli Hao, He Yi, Zhiyue Liu, Yumin Gao, Yuki Eshita, Wenfang Guo, Hairong Zhang, Juan Sun

**Affiliations:** 1 Department of Public Health, Inner Mongolia Medical University, Huhhot, Inner Mongolia Autonomous Region China; 2 Oita University, Faculty of Medicine, Oita, Japan

**Keywords:** physical fitness test, gender, medical students

## Abstract

**Introduction:**

The aim of present study was to investigate gender differences in physical fitness indexes in regard to BMI (body mass index) levels among Inner Mongolia medical students in China.

**Methods:**

Data on participant characteristics came from basic information contained in the school database. Physical fitness indexes including BMI, vital capacity index, sidestep test, and standing long jump, were conducted.

**Results:**

Female students had a higher rate of normal weight than those of males. The obesity rate of males was 5 times higher compared to females. Compared with male students, female students had a higher pass rate in vital capacity index, sidestep and standing long jump. Females were higher 17% than males in the pass rate of the sidestep test. Males performed better than females in the standing long jump. In both the malnutrition and normal weight group, the pass rate of the 3 physical fitness indexes for both male and female students was higher than obese group. The not pass rate was higher than pass rate both male and female students in the vital capacity index in the obese group.

**Discussion:**

Males had a poor physical fitness level compared with females. Male students may be more likely to spend more time using computers and it will cut down the time of participating in physical activities. So, in our university, more attention should pay on physical education, especially for males.

## 1. Introduction

Enhancement of the quality of human life is driven by many things, including searching for cures to diseases, modifying human performance over a human being’s lifespan, and the generation of new knowledge (William, 2000). The survival of our ancestors was in part determined by physical fitness (O’keefe, Vogel, Lavie, & Cordain, 2010), and today health-related physical fitness is defined as fitness related to some aspect of health. It is also regarded as a major marker of health status at any age. It comprises 4 main domains including strength and endurance of skeletal muscles, joint flexibility, body composition, and cardio respiratory endurance (Haskell, & Kiernan, 2000). However, the measurement of physical fitness is influenced by many factors. For example, total body fat, habitual physical activity, and socioeconomic status have been independently correlated to physical fitness (Jiménez et al., 2010). In the category of total body fat, obesity is of most concern, and is measured in proxy fashion in large cross-sectional surveys by elevated body mass index (BMI) (Jonathan, IVan, & Jan, 2011).

In today’s world where the ‘‘infomercial’’ is dominant, and everything works fast, scientists working in the area of sports and exercise have a responsibility to provide the world with factual data and to move the field forward (William, 2000) and several epidemiological studies have measured fitness levels using different methodologies to understand the secular trends and their relation to health(Marques-Vidal, Marcelino, Ravasco, Oliveira, & Paccaud, 2010; Nielsen, & Andersen, 2003; Sassen, Kok, Schaalma, Kiers, & Vanhees, 2010). However, maintaining an appropriate level of health-related physical and reducing the risk of disease and injury fitness requires that individuals participate in physical activity (Gulati et al., 2003). Physical education (PE) at all levels has a vital role in establishing positive lifestyle behaviors and particularly in improving fitness in children and adolescents (Sandor et al., 2010). In regard to the latter, school-based PE interventions, including modified PE classes, generally with more classroom time and more moderate-to-vigorous PA (Physical activity) are key (Kahn et al., 2002). Therefore, based on our previous study (Bian et al., 2012), and including gender as an important factor, our aim was to further explore the gender differences of physical fitness tests among medical students in Inner Mongolia, China.

## 2. Methods

### 2.1 Experimental Approach to the Problem

This investigation was based on a cross-sectional survey that included physical fitness tests for medical students in Inner Mongolia. All students participated in 4 tests, which used the same strength and conditioning apparatus. The tests included BMI, vital capacity index, sidestep test, and standing long jump.

### 2.2 Subjects

The study was approved by the Ethics Committee of Inner Mongolia. In this study we chose students from the Inner Mongolia Medical University, who participated in physical fitness tests. They are the students who were enrolled in 2008, 2009, and 2010 respectively.

### 2.3 Procedures

Students performed four physical fitness tests in a gymnasium. Test steps were as follows: Measurement of height and weight used HHTC/ST model apparatus (Beijing Huaxia Huihai Technology Limited Company). Students stood on a base plate barefooted and while wearing light clothes pressed a button and the height and weight showed on the screen. Body mass index (BMI) was calculated as weight (kg)/height^2^ (m). The BMI was classified into five levels: obese (50 points), overweight (60 points), appropriate weight (100 points), underweight (60 points) and malnutrition (50 points) from the range of standard height and weight using the *College Student Physical Health Standard*. In order to distinguish the same scores (points) weight groups, we defined obese as 50^+^, overweight as 60^+^, underweight as 60^-^, malnutrition as 50^-^ for both male and female.

Physiology was tested using the vital capacity index. The measurement of vital capacity used the XF495-KDL model apparatus (Beijing Baiwan Electronic Technological Apparatus Center). Students put their mouth into the blowpipe and stood before the apparatus to hold the handle properly. Then students pressed the button, took a deep breath, and completely exhaled. The apparatus calculated the maximal breathing capacity automatically. The vital capacity index is calculated as: Vital capacity (ml)/weight index (kg).

Lower limb muscle strength and endurance was tested using the sidestep test (TDK-2 intelligent apparatus, Ningbo Jingbei Tiandikuan Electronic Product Manufacturer). Male students used a 40 cm-high footstep and female students a 35 cm-high footstep to do the up-and-down movement. Students performed up-and-down movements for about 3 minutes (90 repetitions of up-and-down movements) in rhythm to music. Afterward, students sat down and then placed a clip on the middle finger. Pulse rate showed on the screen after the test. Pulse measurement times were taken at 3 times: 1 minute to 1 minute 30 seconds; 2 minutes to 2 minutes 30 seconds; and 3 minutes 30 seconds after the movements. The sidestep test index is equal to duration of up-and-down movements (s)*100/2*(sum of pulse measurement 3 times).

Lower limb explosive strength was tested using the standing long jump measured in meters (81/CVS-1E model apparatus, Beijing Zhongxi Yuanda Technological Company). Students separated their legs naturally, pressed the button, then jumped from their standing point; the result was displayed on the screen.

All physical fitness measurements were classified according to the College Student Physical Health Standard (College student physical health standard, 2008) established by Ministry of Education of China and the General Administration of Sport of China (for classification of vital capacity index, sidestep test index, and standing long jump were classified, see Table 1; Arabic number order is from high to low level with lower numbers better.

**Table 1:** College student physical fitness standards in China

	Level	Vital Capacity Index	Sidestep Test Index	Standing Long Jump (m)
Male			
Level 2	Pass	>54	>45	>2.12
	Not pass	<54	<45	<2.12
Level 4	Excellent	78-84	67-82	2.58-2.66
	Good	68-77	53-65	2.38-2.57
	Normal	55-66	46-52	2.14-2.37
	Poor	<54	<45	<2.12

Female				
Level 2	Pass	>42	>41	>1.56
	Not pass	<42	<41	<1.56
Level 4	Excellent	64-70	60-78	1.99-2.07
	Good	54-63	49-59	1.79-1.98
	Normal	43-53	42-48	1.58-1.76
	Poor	<42	<41	<1.56

In order to learn more about the results of the physical fitness test we investigated time spent by students playing on the computer.

### 2.4 Statistical Analyses

Wilcoxon tests (Z) were used to analyze the differences between male and females for 3 BMI groups: 3 scores of 60^+^ (overweight), 100 (appropriate weight), 60^-^ (underweight) consolidated from 5 BMI scores. Chi square test were used to compare differences between male and females in vital capacity index, sidestep test, and standing long jump at 2 levels. SPSS (SPSS, Inc., Chicago, IL, v.13 was used to carry out statistical analysis with a significance level of p < 0.05.

## 3. Results

A total of 6797 students participated in this study of which 1954 were male and 4833 were female, of whom 89.3% were normal weight. Pass rates for vital capacity index, sidestep index, and standing long jump were 81.5%, 92.0%, and 80.7%, respectively. Of students using a computer every day was 41.6% were male and 14.0% female; 18.1% of males used a computer 3 hours or more per day compared to 9.8% for females.

Significant gender differences were observed in physical fitness tests (Table 2). Female students had a higher prevalence of normal weight compared to males while the obesity prevalence for males was 5 times higher compared to females. In addition, compared to male students, females had a higher pass rate in vital capacity index, sidestep, and standing long jump.

**Table 2:** Physical fitness test by gender

Characteristic	Male		Female	

	n	%	n	%
BMI*			
Malnutrition	50	2.56	99	2.0
Normal weight	1515	77.53	4552	94.0
Obese	389	19.91	192	4.0
Vital capacity index**			
Pass	1524	78	4012	82.8
Not Pass	430	22	831	17.2
Sidestep test***			
Pass	1711	87.6	4544	89.5
Not Pass	243	12.4	299	10.5
Standing long jump****			
Pass	1547	79.2	3935	81.3
Not Pass	407	20.8	906	18.7

* Wilcoxon rank sum test, Z=18.205, p <.001

** Chi square, 21.65, p <.001

*** Chi square, 4.255, p = .039

**** chi square, 3.99, p = .046.

**Figure 1:**
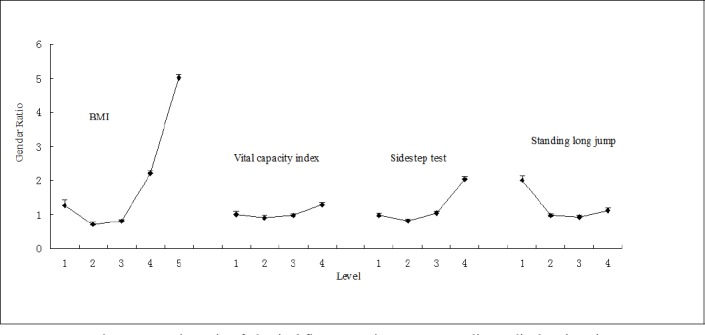
Gender ratio of physical fitness test in Inner Mongolia Medical University

The gender ratio for BMI showed a rising linear trend from the third level to fifth level with the gender ratio for obesity (fifth level) being the highest (Figure 1). Males were similar to females in vital capacity index but had a higher poor rate in the sidestep test and were twice as good in the standing long jump.

Males categorized with malnutrition were similar to the same group of females in vital capacity index and standing long jump (Table 3) but females were 17% higher compared to males in the pass rate of the sidestep test. Normal weight males were similar to the same females in the 3 physical fitness indexes. In contrast, in obese group, the not pass rate of vital capacity index was higher than the pass rate for both male and female students. The pass rate of the 3 physical fitness tests in the malnutrition and normal weight group was higher than those in the obese group for both males and females students.

**Table 3:** Physical fitness test by BMI and gender

Gender	Test	Malnutrition n (%)	Normal n (%)	Obese n (%)
	Vital capacity index			
	Pass	48 (94.1)	1375 (85.1)	194 (47.3)
	Not Pass	3(5.9)	133(8.2)	216(52.7)
	Sidestep test			
Male	Pass	480 (77.2)	1073 (93.2)	328(80.0)
	Not Pass	142 (22.8)	78 (6.8)	82 (20.0)
	Standing long jump			
	Pass	37 (72.6)	1321 (81.8)	285 (69.5)
	Not Pass	14 (27.5)	294(18.2)	125 (30.5)

	Vital capacity index			
	Pass	97 (90.7)	4049 (84.2)	100 (48.3)
	Not Pass	10(9.4)	758(15.8)	107(51.7)
	Sidestep test			
Female	Pass	101 (94.4)	4514 (93.9)	179(86.5)
	Not Pass	6(5.6)	293 (6.1)	28(13.5)
	Standing long jump			
	Pass	83 (77.6)	3931 (81.8)	146 (70.5)
	Not Pass	24 (22.4)	876(18.2)	61 (29.5)

**Figure 2:**
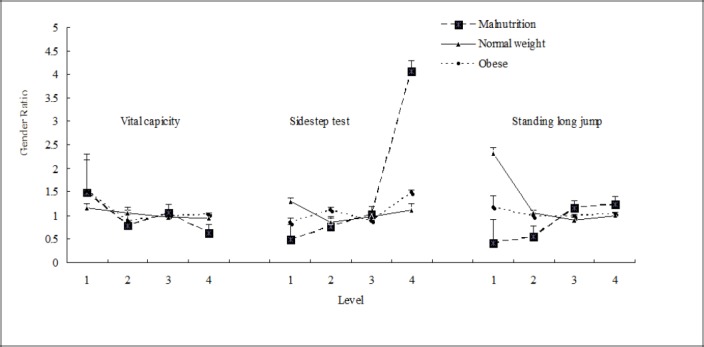
Gender ratio of physical fitness test for BMI in Inner Mongolia Medical University

Males were similar to females in vital capacity index in the 3 BMI groups (Figure 2). Malnutrition males were about 4 times higher than the same group of females at level 4 of the sidestep test. Normal weight males were nearly twice as high compare to females at the excellent (first level) of standing long jump.

## 4. Discussion

Creating a better understanding of human strength and conditioning and advancing practices based upon scientific fact is an important step forward and our challenge was to investigate gender differences in physical fitness and relate those differences to BMI levels.

Our current study shows that males perform worse than females in lower limb muscle strength and endurance. In general, women are capable of longer endurance times compared to men for contractions performed at low to moderate intensities (Hunter & Enoka, 2001). For example, the endurance time of women was longer than that of men when performing an isometric contraction at 20% of maximum with the knee extensor muscles but not at 50 or 80% of maximum (Semmler, Kutzscher, & Enoka, 1999). However, many studies have demonstrated that males have higher strength values than females (Lamarche, Gammage, & Gabriel, 2011; Pangrazi, & Corbin, 1990). Moreover, for maximum voluntary contraction (MVC), males have significantly higher strength than women (Lamarche, Gammage, & Gabriel, 2011). Other studies have shown that boys perform better than girls in explosive strength, endurance of muscles, and speed and that females have lower levels of physical activity and team sports (Kynna, 2011). While in most cases gender-related factors allow males to build more strength than females this may be not always true, and in regard to endurance females may excel more than males. In our study, females participate in more endurance exercises than males in Inner Mongolia.

Males performed better than females in standing long jump. This means that males were better than females in lower limb explosive strength, similar to previous studies (Shang, 2010). The conventional explanation is that compared with girls, boys are more physical active (Nicola, Stuart, & Gareth, 2010) and that boys have greater muscle mass, bone density (Monyeki, Kemper, & Makgae, 2008), and therefore, this trend should continue into young adulthood.

Our current study revealed that both male and female obese students performed worse in physical fitness tests, as observed in other studies in which being overweight or obese first decreased physical exercise capability and then reduced health-related physical fitness (Kovács, Fajcsák, Gábor, & Martos, 2009). Moreover, adolescents at the lowest level of physical fitness are more likely to have high BMI, and a higher number of cardiovascular disease risk factors (Jekal et al., 2009). In our previous study, while we noted that male and female students in the obese category also had a poor physical health status (Bian, 2012) not all obese or overweight subjects are physically unfit. For example, in 2 studies, overweight boys had high physical fitness scores (Monyeki, Neetens, Moss, & Twisk, 2012), and no significant correlation between BMI and fitness test was found in girls (Kwok-Kei et al., 2010).

The findings of our study show that being overweight or obese was more common among male than female students and normal weight was more prevalent among females compared to males in the studied population. In our previous study, the prevalence of obesity was 19.7% in males 4.0% in females (Bian et al., 2012) with many other studies supporting our findings (Najat, Alice, Abbass, & Sandra, 2008; Arngrimsson, Richardsson, Jonsson, & Olafsdottir, 2012).

School curricula, such as sports-meeting and health education lessons, as well as sedentary habits and/or incorrect eating behavior were found to be associated with weight status (Li, Michael, & Hong, 2011; Enza, Patrizia, Gabriele, & Giuseppe, 2009). At our university, male students are more likely to spend more time playing on the computer. Spending more time on the computer is likely to reduce time for taking part in physical activities. This is part of a larger problem in which young adolescents seem to be spending more time watching TV, playing video games, or using personal computers in lieu of participating in physical activity (Thomas, 1999). The lesson, therefore, from an educational viewpoint, is that we need to strengthen physical education curricula, especially for males.

## 5. Conclusion

Males had a poor physical fitness level compared with females’ among Inner Mongolia medical students in China. Obesity impacted on physical fitness indexes. We should pay more attention to physical fitness of males, especially for obese students.
